# Overweight and Obesity: Risk Factors in Calcium Oxalate Stone Disease? 

**DOI:** 10.1155/2012/438707

**Published:** 2012-04-05

**Authors:** Beate Maria Wrobel, Gernot Schubert, Markus Hörmann, Walter Ludwig Strohmaier

**Affiliations:** ^1^Department of Urology and Paediatric Urology, Klinikum Coburg, Ketschendorfer Strasse 33, 96450 Coburg, Germany; ^2^Urinary Stone Laboratory, Vivantes MVZ for Laboratory Diagnostics, Berlin, Germany

## Abstract

*Introduction*. Several studies showed an association of overweight and obesity with calcium oxalate stone disease (CaOx). However, there are no sufficient data on the influence of body weight on the course of the disease and the recurrence rate. *Patients and Methods*. *N* = 100 consecutive stone formers with pure CaOx were studied. Different parameters were investigated. According to the BMI, patients were divided into three groups: (1) BMI ≤ 25; (2) BMI 25.1–30; (3) BMI > 30. *Results*. *N* = 32 patients showed a BMI ≤ 25, *n* = 42 patients showed a BMI of 25.1–30 and *n* = 26 patients showed a BMI ≥ 30. The groups differed significantly concerning BMI (by definition), urine pH, and urine citrate. The recurrence rate was not significantly different. *Discussion*. Our study demonstrated that body weight negatively influences single risk factors in CaOx, but obesity is not a predictor for the risk of recurrence in CaOx.

## 1. Introduction

Several studies showed an association of overweight and obesity with calcium oxalate stone disease (CaOx) [1–3]. There was a correlation between body weight and promoters of CaOx. In general stone disease is often associated with diabetes [4–6]. For example, Maalouf et al. have shown in 2004 that there's an reciprocal correlation between urinary pH and overweight [7]. Other metabolic risk factors are correlated with body weight: urinary pH, uric acid, calcium, citrate, and oxalic acid [8–10]. So far, however, there are no sufficient data on the influence of body weight on the course of the disease and the recurrence rate of CaOx. 

## 2. Patients and Methods

*N* = 100 consecutive stone formers with pure CaOx treated in the Department of Urology and Paediatric Urology at the Klinikum Coburg, Germany, were studied. Stone analysis was performed by polarization microscopy and X-ray diffraction. A detailed history including the number of stone episodes occurring in the past was recorded.

The BMI was calculated after determining body weight and height. Arterial blood pressure (RR) was measured according to the recommendations of the World Hypertension League sitting after 5 minutes at rest. The following parameters were determined in all these patients: Urine pH profiles on three consecutive days at morning (fasting), noon (postprandial) and evening (postprandial). For urine pH measurements, dipsticks were used which allow pH measuring in 0.1 steps (Madaus GmbH, Cologne, Germany). The mean urinary pH was calculated in every patient.

Blood was drawn to measure creatinine (Jaffé reaction, Dade Behring Marburg, Germany), potassium (atomic absorption), calcium (indirect ion sensitive electrode), glucose (postprandially; hexokinase-glucose-6-phosohate dehydrogenase method, Flex Siemens Healthcare Diagnostics Newark, DE, USA), and uric acid (modified uricase method, Dade Behring Marburg, Germany). A 24 h-urine specimen was collected to determine the excretion of citrate (citrate lyase method, Boehringer Mannheim, Germany), creatinine (Jaffé reaction, Dade Behring Marburg, Germany), calcium (indirect ion sensitive electrode), uric acid (modified uricase method, Dade Behring Marburg, Germany), ammonia (modified glutamate dehydrogenase method using NADPH, test kit Ammonia Flex, Dade Int., Newark, DE, USA), and urea (urease-glutamate dehydrogenase, Dade Behring Marburg, Germany) as a marker for protein intake.

According to the BMI, patients were separated into three groups: (1) BMI ≤ 25; (2) BMI 25.1–30; (3) BMI > 30.

In group 1 there were *n* = 23 men and *n* = 10 women in the age of 21–85 years, in group 2 *n* = 27 men and *n* = 14 women in the age of 23–78 years, and in group 3 *n* = 20 men and *n* = 6 women in the age of 27–78 years.

For statistic evaluation, the Kruskal-Wallis test was used. It analyses the statistical significance between three groups (level of significance *P* < 0.05). For box blot graphs, we used the program Prism 3 (GraphPad Software, San Diego, CA, USA). The other figure was made with the use of microsoft excel. Calculations were performed on a personal computer.

## 3. Results

The groups differed significantly concerning BMI (by definition), urine pH, and urine citrate. With regard to the distribution between the sexes the group differed not significantly.

**Figure 1:**
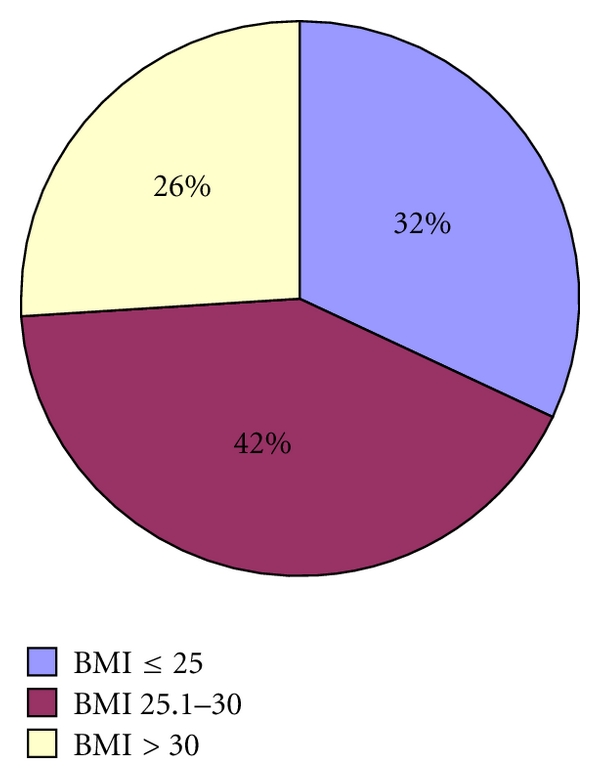
BMI and groups (*P* < 0.001, statistically significant).

*N* = 32 patients (32%) showed a BMI ≤ 25 (group 1) (standard deviation (std) 1.67), *n* = 42 patients (42%) showed a BMI of 25.1–30 (group 2) (std 1.308) and *n* = 26 patients (26%) showed a BMI ≥ 30 (group 3) (std 3.795). Compared with Figure 1.

If you look at urinary pH you can see a higher urinary pH in group 1 with lower BMI than in groups with higher BMI.

**Figure 2:**
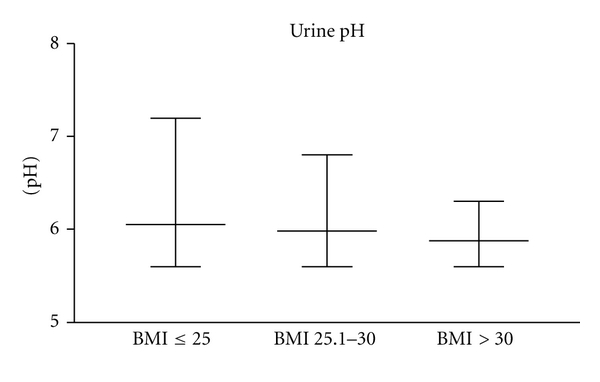
Urine pH in all three groups (*P* = 0.0476, statistically significant).

Group 1 had a median of urine pH of 6.20 (5.6–7.2; std 0.3931), group 2 a median of 6.06 (5.6–6.8; std 0.2879), and group 3 a median of 5.95 (5.6–6.3; std 0.1873). Compared with Figure 2.

Urine citrate excretions also differed significantly.

**Figure 3:**
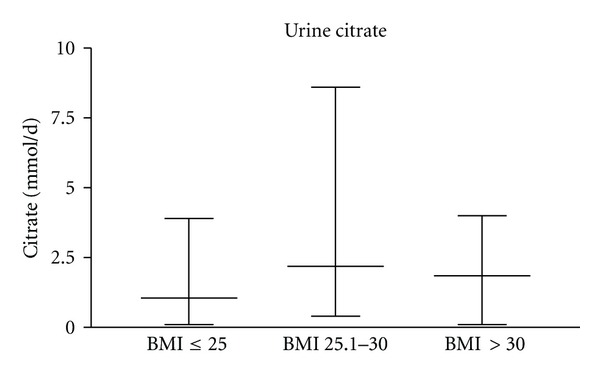
Urine citrate (mmol/d) in all three groups (*P* = 0.0309, statistically significant).

Group 1 had a median of urine citrate of 1.41 mmol/d (0.1–3.9; std 0.9319), group 2 a median of 2.32 mmol/d (0.4–8.6; std 1.622), and group 3 a median of 1.92 mmol/d (0.1–4.0; std 1.096). The highest urine citrate with 8.6 mmol/d could be found in group 2 with a BMI of 25.1–30. Compared with Figure 3.

All the other parameters showed no significant differences. Especially the recurrence rates were not significantly different. There was even a tendency towards a decreasing number of stone episodes with increasing body weight.

**Figure 4:**
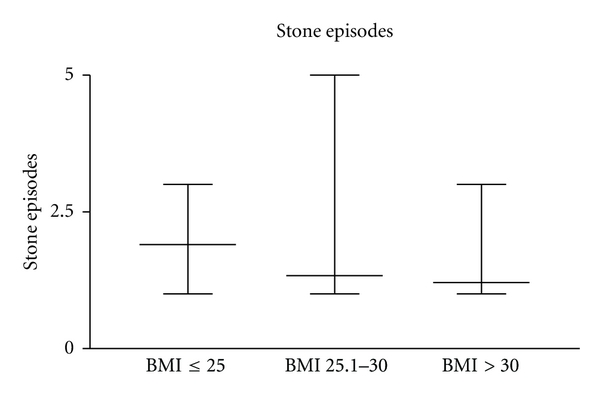
Stone episodes of group 1, 2, and 3 (not significantly different).

Group 1 had a median of 1.183 stone episodes (1–3; std 0.7803), group 2 a median of 1.683 (1–5; std. 1.083), and group 3 a median of 1.423 stone episodes (1–3; std 0.5778). However, the numbers of stone episodes was not significantly different. Compared with Figure 4.

## 4. Discussion

Several studies showed an association of overweight and obesity with calcium oxalate stone disease (CaOx) [1–3, 11, 16]. West et al. described that factors of the metabolic syndrome (included overweight/obesity) are associated with kidney stones [11]. Many of these studies did not differentiate between different stone types, but only looked at a potential association between BMI and the fact whether patients reported a kidney stone in the past. They did not investigate the course of the disease and the recurrence rate, respectively.

There was a correlation between body weight and promoters of CaOx [1–3]. However, when adjusted for multiple covariates, there was no influence of BMI on these risk factors and the calcium oxalate supersaturation [12]. Positive associations between BMI and urinary calcium excretion are due to differences in dietary habits (animal protein and sodium intake) [12].

In general, stone disease is often associated with diabetes, a part of the metabolic syndrome [4–6]. This is in particular true for uric acid lithiasis. If you look at this you can see that reducing insulin resistance by for example, reducing body weight may increase urinary pH and therefore it might be helpful for preventing stone forming [11, 13]. In our population there were only *n* = 7 people with diabetes and we couldn't confirm that reducing body weight and decreasing insulin resistance only will help to increase urinary pH.

We investigated only CaOx stone formers. It is important to separate between different stone compositions. As shown by Taylor and Curhan [12], BMI had differently influenced stone risk factors in CaOx compared with uric acid stone formers. In our population, people with higher BMI (included people with diabetes) and lower urinary pH had a decreased recurrence rate. Therefore, it is not possible to predict the probability of forming stones by measuring urinary pH [14]. 

In our study, most people were overweight or obese (68%). In the general population there are only 49% overweight or obese [15]. Compared to this, there were more obese subjects (BMI ≥ 30) among our CaOx stone formers. This is a higher number than in previous studies [1]. But obviously, this had no impact on the recurrence rate.

Siener et al. [1] and also Taylor et al. [16] wrote that people with higher BMI had an increased risk of kidney stone formation. Sarica et al. described in 2011 that a correct body weight for example could prevent one from forming stones [17, 22].

All these studies did not look at the recurrence rate but only at a self-reported history of kidney stones. We investigated the influence of overweight and obesity not only on fact of having a kidney stone in the past, but also on the course of the disease. There was no influence of body weight on the recurrence rate in our population. People with higher BMI had a decreased recurrence rate. Therefore, obesity is not a predictor for the risk of recurrence in CaOx.

This may be not surprising at second sight. Taylor and Curhan [12] reported that there was no relation between BMI and calcium oxalate supersaturation. They suggested that risk of calcium oxalate stone formation does not increase with increasing body size. They suggested that greater incidence of kidney stones in the obese may be due to an increase in uric acid nephrolithiasis. This is in accordance with our findings that the recurrence rate of calcium oxalate stones does not increase with increasing BMI.

Above that it is very questionable if reducing body weight may positively influence kidney stone forming disease. Maybe eating habits may be more important than reducing body weight.

In our population there were 30 women and 70 men. In the study of Taylor it was written that women were more influenced by obesity and stone forming than men. Maybe this is correct because men were often in our population first stone formers in contrast to women, who had more than one stone in life. Maybe the hormones are important for that, too. Most women were in (pre- or postmenopause).

But in accordance with previous reports body weight negatively influences single risk factors in CaOx [1, 9, 10, 13, 17].

Risk factors for forming stones like for example urinary citrate are often reviewed in studies. There is also written that lower urinary citrate may lead to forming CaOx [18]. In our study this hypothesis could be confirmed, too. There was also like in several studies before a decreased number of forming stones with higher urinary citrate [19]. Therefore, citrate excretion has to be a protective factor against forming stones. Opposed to that, Sikora et al. wrote that in their population most people with CaOx had normal urinary citrate [20]. So only looking at urinary citrate you wouldn't get a predictor for forming stones. 

Urolithiasis is a multifactorial disease [21]. Lifestyle and eating habits have an effect on lithogenic urinary risk factors [22]. Maybe the composition of food has a higher influence on forming stones than body weight. Therefore, a change in food composition might be promising.

In conclusion, our study demonstrated that body weight negatively influences single risk factors in CaOx, but obesity is not a predictor for the risk of recurrence in CaOx.
